# Dietary Zinc Altered the Growth, Serum Biochemical Parameters and Immunity of Juvenile Chinese Soft-Shelled Turtle (*Pelodiscus sinensis*)

**DOI:** 10.1155/anu/5545197

**Published:** 2025-06-13

**Authors:** Hongyan Kou, Dehui Pan, Junru Hu, Xueting Liu, Yiwen Zhao, Guangren Ye, Yutao Miao

**Affiliations:** ^1^Guangzhou Key Laboratory of Aquatic Animal Diseases and Waterfowl Breeding, College of Animal Sciences and Technology, Zhongkai University of Agriculture and Engineering, Guangzhou 510225, Guangdong, China; ^2^Guangdong Key Laboratory of Animal Breeding and Nutrition, Key Laboratory of Animal Nutrition and Feed Science in South China of Ministry of Agriculture and Rural Affairs, Institute of Animal Science, Guangdong Academy of Agricultural Sciences, Guangzhou 510640, China; ^3^Key Laboratory of Ecology and Environment Science in Guangdong Higher Education, Guangdong Provincial Key Laboratory for Healthy and Safe Aquaculture, College of Life Science, South China Normal University, Guangzhou 510631, China

**Keywords:** biochemical indices, feed utilization, immunity, SGR, soft-shelled turtle, zinc

## Abstract

Zinc (Zn) is a kind of critical mineral element for aquaculture and play an important role in growth performance and immunity. Hence this study was aimed to assess alterations in specific growth rate (SGR), feed utilization, activities of digestive enzymes, serum biochemical indexes, the quantity and expression of immunoglobulin M (IgM) in juvenile soft-shelled turtle, *Pelodiscus sinensis*, response to dietary gradient Zn levels. Fish meal-based diets were supplemented with 0, 10, 20, 30, 40, and 50 mg/kg Zn, the analyzed dietary Zn contents were 35.43, 46.23, 55.38, 66.74, 75.06, and 85.24 mg/kg Zn, respectively. ZnSO_4_ · 7H_2_O was used as the Zn source. Turtles (weighing ~4 g) were divided into six groups and were stocked for 12 weeks. The results indicated that SGR, pepsin activity in the stomach, intestinal alpha-amylase and lipase activities, total protein content, acid phosphatase (ACP), and alkaline phosphatase (AKP)activities, the contents and mRNA of IgM and insulin-like growth factor-I (IGF-I) enhanced with dietary Zn inclusions to 55.38 or 66.74 mg/kg and then diminished with increasing Zn contents. Escalating Zn levels to 66.74 mg/kg lowered the feed conversion ratio (FCR), serum aspartate aminotransferase (AST), alanine transaminase (ALT) activities, urea nitrogen, triglyceride (TG), cholesterol, and myeloperoxidases (MPO) activity, beyond which they improved. Insufficient or extra Zn depressed the SGR, reduced the feed utilization and digestive enzyme activities, changed the biochemical indicators, and depressed the immunity. The recommended level of Zn in juvenile *P. sinensis* is 63.75, 61.25, and 61.20 mg/kg diet, derived from analysis of SGR, serum AST, and ALT activities.

## 1. Introduction

Soft-shelled turtle *Pelodiscus sinensis* is cultivated on a large scale in Asian countries, including China, Korea, etc., and is currently a great concern among researchers [1]. Because of its good meat quality, high protein content, and low lipid content [2], it has become an important aquaculture species and has also been widely used in cosmetics and medicinal materials.

Zinc (Zn) is a necessary trace mineral that has an important structural, catalytic, and regulatory impact on many biological functions [3]. Zn serves as an essential cofactor for numerous enzymatic systems, including digestive enzymes, DNA polymerase, protein synthase, and lipid metabolic enzymes. Through these roles, Zn orchestrates the regulation of protein, lipid, and carbohydrate metabolism, as well as immune function. Its multifaceted mechanisms of action include: (1) direct enzymatic activation (as a metalloenzyme cofactor and Zn finger protein component); (2) modulation of key signaling pathways (e.g., insulin signaling cascades); (3) regulation of gene expression (e.g., Cu/Zn-SOD transcription); and (4) maintenance of macromolecular structural stability [4]. In multiple stressed *Pangasianodon hypophthalmus*, dietary Zn enhances growth via growth hormone receptor 1 (GHR1), growth hormone receptor β (GHRβ), and growth hormone (GH) upregulation while boosting immunity through heat shock protein 70 (HSP70) induction, NF-*κ*B pathway, and inducible nitric oxide synthase (iNOS) modulation [5]. Zn is related to bone formation [6] and exerts neuroprotective effects against arsenic-induced cerebral damage in the brain of carp through multiple mechanisms: (1) enhancing endogenous antioxidant capacity via upregulation of catalase and Cu/Zn-SOD activities, (2) activation of the Nrf2/Keap1 antioxidant signaling pathway, and (3) mitigation of endoplasmic reticulum stress (ERS) - a critical pathological mechanism in arsenic-mediated neurotoxicity [7]. Waterborne Zn cannot meet the demand of aquatic animals for Zn [8]. Low levels of Zn can hinder growth, reduce weight gain and survival rates, reduce the Zn content in tissues, and cause skin lesions, etc. [9, 10]. Zn deficiency triggers inflammatory and apoptosis in hepatocytes of grass carp through NF-*κ*B signaling pathway, the regulation of Zn-finger protein A20 and suppressing the formation of the Fas/FADD death-inducing signaling complex (DISC) [11]. However, extra dietary Zn may be harmful or toxic to aquatic animals [12, 13]. The effects of dietary Zn on the different species of aquaculture were shown in Table 1. Therefore, given the importance of Zn, determining the dietary Zn content is of great significance for the farming of Chinese soft-shelled turtles.

Until now, there is no data about the alteration of digestive enzyme activity, serum biochemical indices, the immunoglobulin M (IgM) and IGF-I of *P. sinensis* due to gradient dietary Zn levels. Therefore, this work aimed to estimate the influence of dietary Zn on feed utilization, specific growth rate (SGR), digestive enzyme activities, serum biochemical parameters (total protein content, urea nitrogen, glucose, triglyceride [TG], cholesterol), immunity related parameters (alanine transaminase [ALT], aspartate aminotransferase [AST], acid phosphatase [ACP], [AKP], Myeloperoxidases [MPO]), the content and gene expression of IgM and IGF-I in juvenile *P. sinensis*.

## 2. Materials and Methods

### 2.1. Animals

Soft-shelled turtles (weighing around 4 g) were purchased from a private farm in Guangzhou, China. A basal diet was fed twice a day during a 2-week acclimation. Animal treatment and experimental procedure were applied to the Institution of Animal Care and Use Committee of Zhongkai University of Agriculture and Engineering (ZK20190187).

### 2.2. Experiment Diets

The basic diets were designed based on our previous experimental formula [6]. The basal diets with 32.45% protein were provided with 0, 10, 20, 30, 40, and 50 mg/kg Zn serving ZnSO_4_ · 7H_2_O as the Zn source. The final Zn concentrations were 35.43, 46.23, 55.38, 66.74, 75.06, and 85.24 mg/kg, respectively. The experimental diets were maintained at −20°C.

### 2.3. Feeding Trial

This experiment was conducted in Zhongkai University of Agriculture and Engineering. In total, 360 healthy turtles were unselectively assigned into six groups. Each group had 60 turtles. One turtle was stocked in each plastic cylindrical container (30 cm D × 18 cm H) to stop from fighting. The rearing water temperature were kept at 27 ± 2°C, pH = 7.9, and Zn concentrations in the water were 10 μg L^−1^, respectively. The diets were blended with water (1:1.2) to form a dough before feeding to the turtles and fed twice (08:30 and 16:30 h) every day during the 12-week feeding trial.

### 2.4. Analysis and Measurement

#### 2.4.1. Calculations

The following parameters were calculated:

  $$\begin{array}{c} \text{SGR }\left(\%d^{-1}\right)=100\times \left(\text{Ln}\left(\text{final body weight},g\right)-\text{Ln}\left(\text{initial body weight},g\right)\right)/\left(\text{days of the feeding trial}\right) \end{array}$$

  $$\begin{array}{c} \text{Feed conversion ratio }\left(\text{FCR}\right)=\text{dry diet fed}\left(g\right)/\text{weight gain}\left(g\right). \end{array}$$

#### 2.4.2. Sample Collection

The turtles were starved for 1 day after the 12-week breeding trial. Ten turtles from each treatment were decapitated. The blood was taken and centrifuged (1075 × *g*, 4°C) for 10 min to acquire serum. The livers, entire stomachs, intestines and spleen of nine turtles from each group were dissected and preserved at −80°C for later measurement.

#### 2.4.3. Determination of Digestive Enzyme Activities

The sampled stomach and intestines were weighed, then homogenized with cold saltwater (0.75%) at the ratio of 1:5 (*w*/*v*). After centrifugated (1800 × *g*, 4°C) for 10 min, the supernatants were maintained at −20°C for later measurement.

The pepsin activity in the stomach, intestinal activities of alpha-amylase and lipase were analyzed by the colorimetric method and followed the instructions (Jiancheng Bioengineering Institute, Nanjing, China) according to our previous method [23].

#### 2.4.4. Measurement of Serum Parameters

According to the method of Liu et al. [24], Serum activities of AKP, ACP, AST, ALT, total protein, cholesterol, glucose, urea nitrogen, and TG were tested following the relevant kits (Jiancheng Institute of Biotechnology, Nanjing, China).

#### 2.4.5. Test of IgM

The IgM content in serum was determined following the analytical relevant kits (Jiancheng Institute of Biotechnology, Nanjing, China) according to the method described by Liu et al. [25].

#### 2.4.6. Determination of Gene Expressions of IgM and IGF-I in the Liver

The primers encoding for IgM and IGF-I are presented in Table 2. 18S and β-actin was used as the housekeeping genes of IgM and IGF-1, respectively. The procedures were performed following previously described methods [26]. Briefly, the total RNA was then extracted from the samples using an RNAiso Plus Kit (Takara, Dalian, China). After the RNA was reverse transcribed into cDNA, it was used for the amplification with a SYBR Prime Script RT-PCR Kit II (Takara, Dalian, China) by an ABI 7500 real-time PCR machine (Applied Biosystems, Waltham, USA). The gene mRNA expression was calculated by 2^−ΔΔCt^ method.

### 2.5. Statistical Analysis

The findings were processed by one-way ANOVA and Tukey's multiple range test. All the data were shown as mean ± standard deviation and analyzed with SPSS 23.0. Analysis with quadratic regression and broken-line models were performed to assess the relationships between dietary Zn and SGR, serum ALT and AST [27].

## 3. Results

### 3.1. Growth

**Figure 1:**
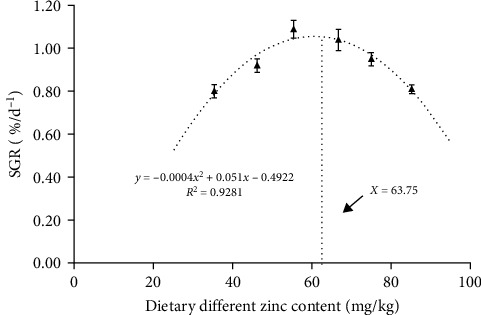
The relation between specific growth rate and zinc contents in the diets for juvenile soft-shelled turtles. Values are presented as means ± SD (*n* = 60). *X* indicates dietary zinc content.

As presented in Table 3, the SGR was enhanced with a proportional elevation in dietary Zn until the 66.74 mg/kg group. No significant changes were found in the 55.38 or 66.74 groups (*p* > 0.05). The SGRs in the 46.23 and 75.06 mg/kg treatments were only second to that of the highest group. Groups supplemented with dietary Zn of 35.43 and 85.24 mg/kg presented the lowest SGR. The suitable inclusion of dietary Zn estimated by SGR was measured by quadratic regression analysis. The maximum was 63.75 mg/kg (Figure 1). The groups with 55.38 and 66.74 mg/kg presented the minimum feed conversion ratio (FCR), while no significant variation was detected among other experimental groups (Table 3).

### 3.2. Activities of Digestive Enzyme

**Figure 2:**
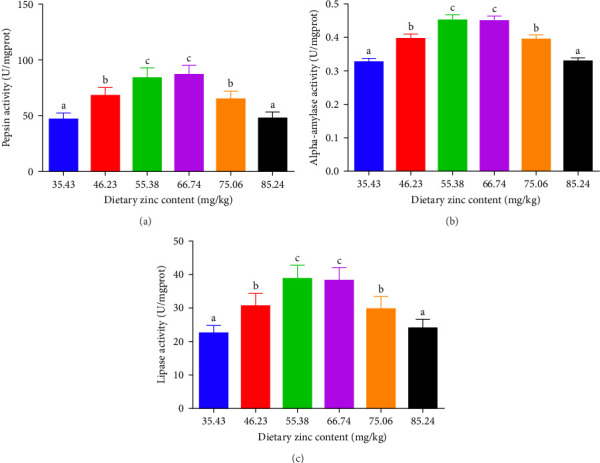
Impact of dietary gradient zinc contents on intestinal digestive activity of juvenile soft-shelled turtles fed diets for 12 weeks. (A–C) Correspond to pepsin activity, alpha-amylase activity, and lipase activity, respectively. Values are presented as means ± SD (*n* = 9). Vertical bar charts sharing diverse letters indicated evident variations among treatments (*p* < 0.05). Different lowercase letters (a, b, and c) above the bars indicate statistically significant differences (*p* < 0.05). Bars sharing the same letter are not significantly different.

Pepsin activity of the stomach, intestinal alpha-amylase, and lipase activities of *P*. *sinensis* were predicted as shown in Figure 2. Pepsin activity was elevated by up to 66.74 mg Zn/kg with escalating Zn levels and then decreased at higher dietary Zn. No statistical variation was observed between 46.23 or 75.06 mg/kg group (*p* > 0.05) (Figure 2A). Intestinal alpha-amylase and lipase activities presented a similar trend to that of pepsin activity. The groups with 55.38 and 66.74 mg/kg had the maximum values (*p* < 0.05) (Figure 2B,C).

### 3.3. Serum Parameters

Total protein, glucose, total cholesterol (TC), urea nitrogen, and TG of *P. sinensis* fed gradient Zn proportions are predicted in Table 4. As the Zn content in the feed increased, the serum total protein content initially rose until reaching a Zn level of 66.74 mg/kg, beyond which further Zn supplementation resulted in a gradual decline in total protein levels. The total protein content of 66.74 mg/kg group reached the maximum value. The serum urea nitrogen significantly diminished with the improvement of Zn levels. When the Zn levels in feed were 55.38 and 66.74 mg/kg, the content of serum urea nitrogen reached the lowest value, and then gradually increased thereafter. With the improvement of dietary Zn, the glucose content gradually decreased to 55.38 and 66.74 mg/kg, and then remained stable. The glucose content reached a minimum value in both the 55.38 and 66.74 mg/kg treatments. TG content progressively reduced with the improvement in dietary Zn. When Zn contents in the feed were 55.38 and 66.74 mg/kg, it reached the minimum, and then gradually increased when dietary Zn provision was higher. The content of TC is similar to that of TG. With the improvement of Zn levels, the content of TC progressively reduced to 66.74 mg/kg and then gradually enhanced after reaching the minimum value.

**Figure 3:**
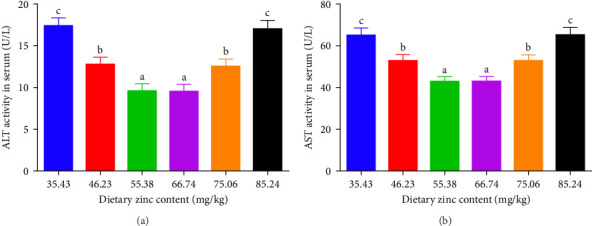
Impact of dietary gradient zinc contents on serum aminotransferase activity of juvenile soft-shelled turtles for 12 weeks. (A) and (B) Correspond to ALT, and AST, respectively. Values are presented as means ± SD (*n* = 5). Vertical bar charts sharing diverse letters indicated evident variations among groups (*p* < 0.05). ALT, alanine transaminase activity; AST, aspartate aminotransferase activity.

**Figure 4:**
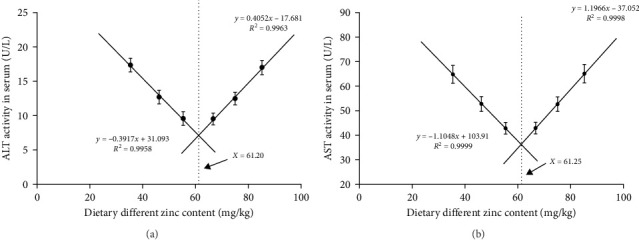
The correlation between serum aminotransferase activity and dietary zinc contents of juvenile soft-shelled turtles. (A) and (B) Correspond to ALT, and AST, respectively. Values are presented as means ± SD (*n* = 5). ALT, alanine transaminase activity; AST, aspartate aminotransferase activity. *X* indicates dietary zinc content.

The inclusions of Zn in the diets increasing from 35.43 to 55.38 mg/kg diminished the ALT at first and then it was elevated to a higher proportion (Figure 3A). The ALT activity of the 55.38 mg/kg group presented the minimum value. The AST decreased with an elevation in the dietary Zn inclusions until 66.74 mg Zn/kg, beyond this it increased at higher Zn levels (Figure 3B). Analysis of broken-line models were performed to estimate the relations between AST and ALT activity against dietary Zn proportions. Based on the results, the recommended dietary Zn for AST and ALT activity of juvenile *P. sinensis* were 61.25 and 61.20 mg/kg, respectively (Figure 4A,B).

### 3.4. Immunity Related Parameters

**Figure 5:**
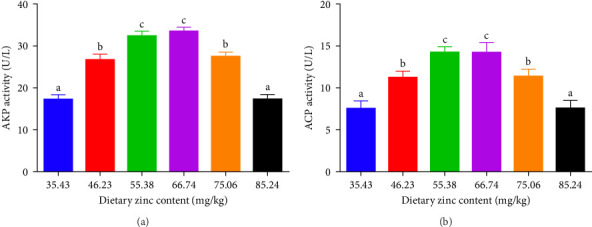
Impact of dietary gradient zinc contents on serum phosphatase enzymes of juvenile soft-shelled turtles for 12 weeks. (A and B) Correspond to AKP, and ACP, respectively. Values are presented as means ± SD (*n* = 5). Vertical bar charts sharing diverse letters indicated evident variations among groups (*p* < 0.05). AKP, alkaline phosphatase; ACP, acid phosphatase. Different lowercase letters (a, b, and c) above the bars indicate statistically significant differences (*p* < 0.05). Bars sharing the same letter are not significantly different.

**Figure 6:**
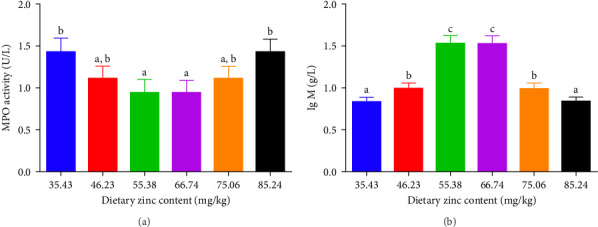
Impact of dietary gradient zinc levels on the immunity indicator of juvenile soft-shelled turtles for 12 weeks. (A and B) Correspond to MPO, and IgM, respectively. Values are presented as means ± SD (*n* = 5). Vertical bars sharing different letters indicated evident variations among groups (*p* < 0.05). IgM, immunoglobulin M; MPO, myeloperoxidases. Different lowercase letters (a, b, and c) above the bars indicate statistically significant differences (*p* < 0.05). Bars sharing the same letter are not significantly different.

ACP, AKP, and MPO activities in the serum of *P. sinensis* are shown in Figures 5 and 6. The ACP (Figure 5B) and AKP (Figure 5A) activity increased up to 66.74 mg/kg and then decreased. No statistical variation was observed in between 55.38 and 66.74 mg/kg groups. MPO activity decreased with elevating Zn levels up to the 55.38 mg/kg and then improved (Figure 6).

### 3.5. The Quantity and Expression of IgM

**Figure 7:**
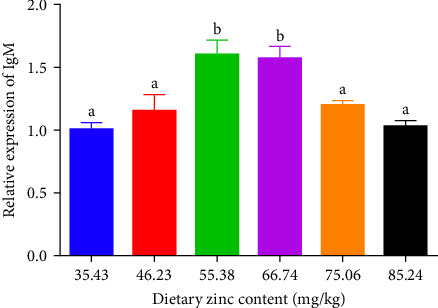
Impact of dietary gradient zinc levels relative expression of IgM in juvenile soft-shelled turtles for 12 weeks. Values are presented as means ± SD (*n* = 9). Vertical bars sharing different letters presented evident variations among groups (*p* < 0.05). IgM, immunoglobulin M. Different lowercase letters (a and b) above the bars indicate statistically significant differences (*p* < 0.05). Bars sharing the same letter are not significantly different.

The quantity and expression of IgM in *P. sinensis* are shown in Figure 7. The quantity of IgM in *P. sinensis* improved profoundly up to 66.74 mg/kg and subsequently diminished with escalating dietary Zn contents. The lowest level of IgM was seen in 35.43 and 85.24 mg/kg treatments (Figure 7). The expression of IgM in *P. sinensis* was improved gradually with elevating Zn levels up to 66.74 mg/kg and then downregulated with further increasing contents. No significant differences were occurred among the 35.43, 46.23, 75.06, or 85.24 mg/kg treatments (*p* > 0.05) (Figure 7).

### 3.6. Gene Expressions of IGF-I in the Liver

**Figure 8:**
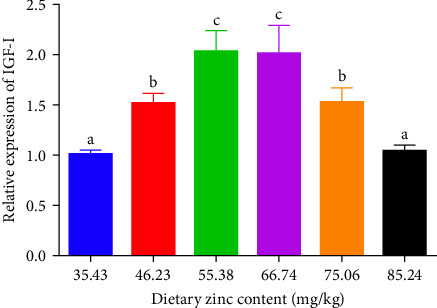
Impact of dietary gradient zinc levels relative expression of IGF-I in juvenile soft-shelled turtles for 12 weeks. Values are presented as means ± SD (*n* = 9). Vertical bars sharing different letters presented evident variations among groups (*p* < 0.05). IGF-I, insulin-like growth factor-I. Different lowercase letters (a, b, and c) above the bars indicate statistically significant differences (*p* < 0.05). Bars sharing the same letter are not significantly different.

The gene expression of IGF-I in the liver of *P. sinensis* are shown in Figure 8. The mRNA expression of IGF-1 was upregulated with increasing dietary Zn levels up to 55.38 mg/kg, and then downregulated at higher Zn levels. No statistical difference was observed between 55.38 and 66.74 mg/kg.

## 4. Discussion

Growth is a critical parameter for the determination of nutritional status. Optimal Zn levels improved the SGR of the turtles. Similarly, Zn supplementation promoted the SGR of Yellow catfish, *Pelteobagrus fulvidraco* [14], gilthead seabream, *S. aurata*, larvae [15], Nile tilapia, *O. niloticus* [16, 21], largemouth bass, *M. salmoides* [18, 19], fingerling *H. fossilis* [22], spotted sea bass [28], and rohu [29]. The SGR of Asian catfish, *C. batrachus* [17] and juvenile Mori, *C. mrigala* [20] increased to 40 mg/kg Zn and then leveled off. Contrary to our findings, dietary Zn did not affect the SGR of *P. sinensis* [26]. The discrepancy may be due to different dietary ingredients and variations in the culture periods. The recommended dietary Zn level for *P. sinensis* is 63.75 mg/kg based on SGR. The Zn requirement is higher than the values (42–46 mg/kg) in previous work [26]. The reason for the differences may be that the antinutrition component (phytate) of dietary ingredients prevents Zn availability in aquatic animals [30]. FCR is a sensitive parameter for estimating the use of feed [31]. With the concentrations of dietary Zn up to 66.74 mg/kg, the FCR diminished and thereafter improved with elevating dietary Zn. Optimal Zn levels decreased the FCR of the *P. sinensis*. This finding parallels well with a study on yellow catfish [14], spotted sea bass [28], and grouper [32]. Contrary to this result, dietary Zn does not influence the FCR of common carp [30], or soft-shelled turtles [26]. Findings may differ if the species, feed ingredients, and culture time are different.

The growth of aquatic animals is related to digestion and absorption. The capability of aquatic animals to acquire nutrition is associated with digestive enzyme activities [33]. Usually, Zn absorption in animals is limited or different depending on the different sections of the gastrointestinal tract and the different ages of the experimental species [34]. The level of Zn in dietary ingredients or natural feed is not high enough to meet the requirements of aquatic species [18]. Our finding demonstrated that optimal dietary Zn levels promoted the digestive enzyme activities in the turtles. The reduced digestive enzyme activities presented in 75.06 and 85.24 mg/kg treatments revealed its toxicity because of excess dietary Zn concentrations. In the absorption process, extra Zn may compete with other bivalent minerals (iron, copper, cadmium, and calcium) in the digestive tract for similar binding sites [10]. Analogous phenomena were presented in other aquatic species, that is giant freshwater prawn [35, 36]. Suitable Zn increased the digestive activities were also found in fingerling *H. fossilis*, while further Zn supplementation had no effect on it [22]. Contrary to our findings, dietary Zn did not have an impact on the activities of digestive enzymes in Nile tilapia [37]. The different species may lead to different results.

Hematological and biochemical parameters are very valuable parameters reflecting the nutrition and physiological disorders of diseases [22]. The serum total protein and albumin of turtles can vary according to diverse physiological status [38]. Supplementation of optimal Zn can improve total protein, whereas extra or deficient Zn diminished the protein content. A lower serum total protein is occurred if the liver is suffering pathological changes and indicates low protein metabolism of aquatic animals [39]. This parallels well with the findings of *Pangasius hypophthalmus* [40], *Lateolabrax maculatus* [31], and *Labeo rohita* (Hamilton) [34].

Glucose, a kind of monosaccharide presented in the blood, is a sensitive parameter to assess physiological stress [41]. Similarly, serum glucose levels of *P. hypophthalmus* [40] and largemouth bass, *M. salmoides* [19] reduced with the addition of Zn nanoparticles (Zn-NPs) and then increased with a further elevation in Zn levels. This can be explained by other results found in this experiment, where an appropriate amount of Zn can promote the increase of IGF-I, thereby promoting the secretion of insulin and inhibiting the increase of blood glucose. High serum glucose content demonstrates that an aquatic animal is under stress and uses its energy deposit extensively [42]. No significant change in glucose levels was found in the study of *Megalobrama amblycephala* [43]. The difference in the findings may be caused by different species and culture time. The culture time may not be long enough to change the serum glucose levels.

TC and TG are critical parameters to estimate lipid metabolism in animals [44]. Similarly, a dietary Zn supplement can reduce the TG of blunt snout bream [45]. The optimal supplementation of Zn can decrease the TC of juvenile *Litopenaeus vannamei* and largemouth bass, *M. salmoides* [19, 46]. It is well known that low TC and TG demonstrate a beneficial effect on health and can help to prevent diseases [47]. Zn may be involved in the lipid metabolism of *P. sinensis*, which need to be studied further.

The urea content is a reliable parameter that reflects the function of the liver and kidneys [48]. In our study, extra or deficient Zn levels improved the urea nitrogen content, which may cause liver or kidney disorders in the turtles. Similar report was found in largemouth bass, *M. salmoides* [19].

Serum AST and ALT are generally considered parameters for the estimation of liver health [49]. Suitable Zn levels decrease serum ALT and AST activities. Deficient or excessive Zn may cause damage to the liver. As we all known, ALT and AST exist principally in livers. When the livers suffered from damage, the permeability of the cell membrane is increased, so serum activities of ALT and AST enhance accordingly [10]. This result paralleled well with the work of Chinese perch (*Siniperca chuatsi*) [44] and beluga sturgeon (*Huso huso*) [50]. Extra or excess Zn may cause liver disorder or damage of aquaculture, so it changes the ALT and AST activities.

AKP and ACP are two crucial parameters in the immune system of aquatic species, which are valuable indicators for evaluating the Zn status [51]. Optimal dietary Zn increased the AKP and ACP activities of juvenile *P. sinensis*, while deficient and extra dietary Zn reduced the AKP and ACP activities. Similar findings were found in *O. niloticus* × *O. aureus* [52], coho salmon (*Oncorhynchus kisutch*) [53], *Eriocheir sinensis* [54], and *Macrobrachium nipponense* [55]. Consistent with our study, the ACP activity is similar to the results found in *L. vannamei* [46] and *E. sinensis* [54]. There are Zn binding sites on the enzymes of AKP and ACP, and an appropriate level of Zn activates the activity of these enzymes.

MPO is a valuable peroxidase reflecting the immunity of aquatic species [56]. In our study, deficient and extra dietary Zn improved the MPO activity. As we all known, deficient and extra dietary Zn may be a nutritional stress for juvenile *P. sinensis*, so it activated the MPO activity. It has been reported that MPO is released during the early immune stage to promote inflammation responses [57]. Znic supplementation also increased the MPO activity of fngerling *H. fossilis* [22].

IgM exists in the serum and mucus of aquatic animals, having a key impact on systemic immunity, as well as mucosal immunity [58]. IgMs are the most common isoforms of Ig in teleosts [59]. Appropriate Zn levels promote the levels of IgM and mRNA expression and improve immunity. The results are similar to previous study on grass carp [60]. However, no significant changes were observed in beluga sturgeon (*H. huso*) [50]. Total serum IgM changes to a certain extent among aquatic animals and also varies according to other parameters, including size, age, gender, environmental factors, season, and vaccination or infection conditions [61]. To our knowledge, upregulation of IgM can help the organism resist pathogen invasion [58]. Therefore, optimal Zn levels improve disease resistance.

Growth is mainly controlled by the Growth hormone/insulin-like growth factor-I (GH/IGF-I) axis. The growth-promoting effect of GH is mainly carried out by IGF-1 [62]. Optimal dietary Zn can upregulate the gene expression of IGF-1, which is consistent with the SGR. This phenomenon can explain the previously discovered results. Optimal Zn can promote the increase of IGF-I, which in turn promotes the increase of GH, thus promoting the growth of juvenile *P. sinensis*. Therefore, compared with the groups with deficient or excessive Zn, the SGR of the optimal Zn group is the highest. Similarly, it was found that higher dietary inorganic Zn can enhance the mRNA expression of IGF-1 in tilapia, *O. niloticus* [63]. Not only does Zn in the feed affect the expression of IGF-1 in aquatic animals, but Zn exposure in aquaculture environments also affects the growth. Excess Zn (Zinc chloride) exposure caused a significant decrease in the expression of IGF-I of zebrafish (*Danio rerio*) at the concentration that hinder the growth [64]. The genes expression of GH was upregulated when the marine medaka *Oryzias melastigma* was exposed to Zn for 0.5 h [65]. Optimal Zn may active the activity of enzymes that can stimulate the secretion of IGF-I.

## 5. Conclusion

Our findings suggest that optimal dietary Zn is necessary for juvenile *P. sinensis* using ZnSO_4_ · 7H_2_O as the Zn source. Insufficient or extra Zn not only depressed the SGR but also decreased feed utilization, digestive enzyme activities, total protein content, the activities of AKP and ACP, the gene expression of IgM and IGF-I, and improved glucose, urea nitrogen, triglyceride, cholesterol, the MPO activity, activities of ALT and AST in serum. The suitable dietary Zn requirement for *P. sinensis* is 63.75, 61.20 and 61.25 mg/kg, based on the analysis using SGR, ALT, and AST as indicators, respectively.

## Figures and Tables

**Table 1:** The effect of zinc on the growth performance in aquaculture.

Species	Zinc form	Optimal zinc levels	The effect of zinc on growth performance	Reference
Yellow catfish *Pelteobagrus fulvidraco*	ZnSO_4_·7 H_2_O	18.69 mg Zn/kg	The fish fed the middle Zn (18.69 mg Zn/kg) diets showed higher growth performance and lower feed conversion rate	Xu et al. [14]
Gilthead seabream (*Sparus aurata*) larvae	ZnSO_4_·7 H_2_O	110–130 mg Zn/kg	Symptoms of Zn deficiency and excess were respectively observed in 79 and 248 mg Zn/kg diet Skeletal anomalies were observe in the 79 mg Zn/kg group	Tseng et al. [15]
Nile tilapia (*Oreochromis niloticus*)	Zinc amino acid complex (Zn-AA)	100 mg Zn-AA	Best growth performance (final weight, final biomass, weight gain, specific growth rate, and feed intake) was found in fish fed with 100 mg Zn-AA kg/diet	Lemos et al. [16]
Asian catfish, *Clarias batrachus*	Nanosized Zn particles (Zn-NPs)	Ranged between 30.4 and 30.5 mg/kg per diet	Fed with Zn-NPs at 40 mg/kg of feed had significantly improved growth performance	Jewel et al [17]
Largemouth bass (*Micropterus salmoides*)	Proteinate Zn	66.57 mg/kg	Weight gain were gradually improved with up to 102.68 mg/kg zinc and decreased at higher levels	Kou et al. [18]
Largemouth bass (*M. salmoides*)	Complex amino acid-chelated zinc	76.99 mg/kg	The specific growth rate (SGR) varied with dietary Zn level in a quadratic model and peaked at the 73.34 mg/kg group	Gu et al. [19]
Juvenile Mori (*Cirrhinus mrigala*)	Zn-gluconate	40 mg/kg of Zn-gluconate supplementation	Weight gain and specific growth rate followed a similar pattern, while weight gain% was enhanced till 40 mg/kg of Zn-gluconate. However, further increase (40–60 mg/kg) had no significant effect on weight gain	Hamid et al. [20]
Nile tilapia (*O. niloticus*)	Green microalga *Pediastrum boryanum* (BIO-ZnNPs)	60 mg (kg)	BIO-ZnNPs inclusion at 60 mg/kg feed showed the most advantage	Zahran et al. [21]
Fingerling (*Heteropneustes fossilis*)	Zinc sulfate heptahydrate	26.82–29.84 mg/kg	Growth performance increased linearly up to 26.74 mg/kg Zn	Zafar et al. [22]

**Table 2:** Primers used for the PCR of immunoglobulin M and IGF-I expression analysis in juvenile soft-shelled turtle.

Name	Sequence
IgM-F	5′-GTCACTGAGAAAGAATGGGAC-3′
IgM-R	5′-GGAGGGCCAGCTGCTCGG-3′
IGF-I-F	5′-TACCTTAACCAATTCTGCCACG-3′
IGF-I-R	5′-AAGCCTCTGTCTCCACATACGA-3′
18S-F	5′-CGTATTGTGCCGCTAGAGGTG-3′
18S-R	5′-CCTCCGACTTTCGTTCTTGATT-3′
β-actin-F	5′-TGATGGACTCAGGTGACGGTGT-3′
β-actin-R	5′-GGCTGTGGTGGTGAAGCTGTAG-3′

Abbreviations: IGF-I, insulin-like growth factor-I; IgM, immunoglobulin M.

**Table 3:** Growth and feed conversion ration of juvenile soft-shelled turtle fed diets with gradient levels of zinc.

Parameters	Different contents of zinc (mg/kg)					
	35.43	46.23	55.38	66.74	75.06	85.24
SGR (% d^−1^)	0.80 ± 0.02^a^	0.92 ± 0.04^b^	1.09 ± 0.05^c^	1.04 ± 0.05^c^	0.95 ± 0.03^b^	0.81 ± 0.02^a^
FCR	1.85 ± 0.32^b^	1.61 ± 0.29^b^	1.18 ± 0.21^a^	1.21 ± 0.24^a^	1.57 ± 0.28^b^	1.75 ± 0.31^b^

*Note*: Values are represented as means ± SD (*n* = 60); different letters (a, b, and c) in a row present evident variation (*p* < 0.05).

Abbreviations: FCR, feed conversion ratio; SGR, specific growth rate.

**Table 4:** Serum parameters of the juvenile soft-shelled turtle fed diets with gradient levels of zinc.

Serum parameters	Different contents of zinc (mg/kg)					
	35.43	46.23	55.38	66.74	75.06	85.24
TP (g/L)	25.63 ± 1.64^a^	27.61 ± 1.47^b^	29.81 ± 2.28^c^	30.51 ± 2.01^c^	27.55 ± 2.50^b^	25.97 ± 1.32^a^
BUN (mmol/L)	0.57 ± 0.11^b^	0.46 ± 0.07^a,b^	0.37 ± 0.06^a^	0.36 ± 0.07^a^	0.49 ± 0.08^a,b^	0.55 ± 0.09^b^
Glucose (mmol/L)	5.84 ± 0.24^a^	6.14 ± 0.32^b^	6.72 ± 0.34^c^	6.85 ± 0.27^c^	6.59 ± 0.25^b,c^	6.46 ± 0.28^b,c^
TG (mmol/L)	15.51 ± 0.76^c^	14.25 ± 0.63^b^	12.98 ± 0.68^a,b^	12.63 ± 0.52^a^	13.70 ± 0.54^a,b^	13.98 ± 0.61^b^
TC (mmol/L)	4.12 ± 0.41^c^	3.37 ± 0.56^b,c^	2.45 ± 0.47^a^	2.58 ± 0.53^a,b^	3.25 ± 0.39^a,b^	4.02 ± 0.37^b,c^

*Note*: Values are represented as means ± SD (*n* = 5); different letters (a, b, and c) in a row present evident variation (*p* < 0.05).

Abbreviations: BUN, blood urea nitrogen; TC, total cholesterol; TG, triglyceride; TP, total protein.

## Data Availability

The data are available from the corresponding author upon reasonable request.

## References

[1] Shang R. S., Man L. M., Wang G. Y., Li M. F., Liu C. J., Li L. S. (2022). Influences of Partial Substitution of Fish Meal With Defatted Black Soldier Fly (*Hermetia illucens*) Larvae Meal in Diets on Growth Performance, Biochemical Parameters, and Body Composition of Juvenile Chinese Soft-Shelled Turtles (*Pelodiscus sinensis*). *Aquaculture Nutrition*.

[2] Hou M., Sun W., Ma Y. (2024). Comparative Analysis for Nutrients, Flavor Compounds, and Lipidome Revealed the Edible Value of Pond-Cultured Male *Pelodiscus sinensis* With Different Ages. *Food Chemistry*.

[3] Kido T., Yanagisawa H., Suka M. (2025). Zinc Deficiency Reduces Intestinal Secretory Immunoglobulin A and Induces Inflammatory Responses via the Gut-Liver Axis. *Immunology*.

[4] Kumar N., Thorat S. T., Patole P. B., Gite A., Kumar T. (2023). Does a Selenium and Zinc Nanoparticles Support Mitigation of Multiple-Stress in Aquaculture?. *Aquaculture*.

[5] Kumar N., Thorat S. T., Chavhan S. R., Reddy K. S. (2024). Potential Role of Dietary Zinc on Gene Regulation of Growth Performance and Immunity in *Pangasianodon hypophthalmus* Against Multiple Stresses. *Aquaculture*.

[6] Philip A. J. P., Fjelldal P. G., Remø S. (2023). Dietary Zinc, Selenium and Water Temperature During Early Seawater Phase Influences the Development of Vertebral Deformities and Cataract in Adult Atlantic Salmon. *Aquaculture*.

[7] Wang W., Zhang Y., Geng X. (2024). Zinc Attenuates Arsenic Overdose-Induced Brain Damage via PERK/ATF6 and TLR/MyD88/NF-*κ*B Pathways. *Comparative Biochemistry and Physiology Part C: Toxicology & Pharmacology*.

[8] Tan B., Mai K. (2001). Zinc Methionine and Zinc Sulfate as Sources of Dietary Zinc for Juvenile Abalone, *Haliotis discus hannai* Ino. *Aquaculture*.

[9] Musharraf M., Khan M. A. (2019). Dietary Zinc Requirement of Fingerling Indian Major Carp, *Labeo rohita* (Hamilton). *Aquaculture*.

[10] Kou H., Hu J., Wang A. L., Pan X., Miao Y., Lin L. (2021). Impacts of Dietary Zinc on Growth Performance, Haematological Indicators, Transaminase Activity and Tissue Trace Mineral Contents of Soft-Shelled Turtle (*Pelodiscus sinensis*). *Aquaculture Nutrition*.

[11] Cui J., Xu T., Lv H., Guo M. Y. (2023). Zinc Deficiency Causes Oxidative Stress, Endoplasmic Reticulum Stress, Apoptosis and Inflammation in Hepatocytes in Grass Carp. *Fish & Shellfish Immunology*.

[12] Kumar N., Krishnani K. K., Kumar P., Singh N. P. (2017). Zinc Nanoparticles Potentiates Thermal Tolerance and Cellular Stress Protection of *Pangasius hypophthalmus* Reared Under Multiple Stressors. *Journal of Thermal Biology*.

[13] Kou H., Hu J., Vijayaraman S. B. (2021). Evaluation of Dietary Zinc on Antioxidant-Related Gene Expression, Antioxidant Capability and Immunity of Soft-Shelled Turtles *Pelodiscus sinensis*. *Fish & Shellfish Immunology*.

[14] Xu X. W., Song C. C., Tan X. Y., Zhong C. C., Luo Z. (2023). Effects of Dietary Zinc (Zn) Levels on Growth Performance, Nutrient Composition, Muscle Development, Antioxidant and Inflammatory Responses in Yellow Catfish Muscle. *Aquaculture Reports*.

[15] Tseng Y., Izquierdo M., Sivagurunathan U., Philip A. J. P., Domínguez D. (2025). Effects of Dietary Zinc on Growth, Bone-Related Genes Expression and Skeletal Anomalies in Gilthead Seabream (*Sparus aurata*) Larvae. *Aquaculture*.

[16] da Pão Lemos C. H., de Oliveira C. P. B., de Oliveira I. C. (2024). Responses to Graded Levels of Zinc Amino Acid Complex in Nile Tilapia (*Oreochromis niloticus*). *Veterinary Research Communications*.

[17] Jewel A. S., Haque A., Akter N. (2024). Effects of Dietary Supplementation of Zn-Nanoparticles on the Growth Performance and Nutritional Quality of Asian Catfish, *Clarias batrachus*. *Frontiers in Sustainable Food Systems*.

[18] Kou H., Liu X., Hu J., Lin G., Zhang Y., Lin L. (2023). Impact of Dietary Zinc on the Growth Performance, Histopathological Analysis, Antioxidant Capability, and Inflammatory Response of Largemouth Bass *Micropterus salmoides*. *Fish & Shellfish Immunology*.

[19] Gu D., Mao X., Azm F. R. A. (2024). Optimal Dietary Zinc Inclusion Improved Growth Performance, Serum Antioxidant Capacity, Immune Status, and Liver Lipid and Glucose Metabolism of Largemouth Bass (*Micropterus salmoides*). *Fish & Shellfish Immunology*.

[20] Hamid Z., Shah S. Z. H., Fatima M., Maryam, Nadeem H., Ali W. (2024). Effects of Zinc-Gluconate Levels on Growth Performance, Whole-Body Composition, and Mineral and Enzyme Activities of Juvenile Mori (*Cirrhinus mrigala*). *Biology and Trace Elements Research*.

[21] Zahran E., Elbahnaswy S., Mansour A. I. A. (2024). Dietary Algal-Sourced Zinc Nanoparticles Promote Growth Performance, Intestinal Integrity, and Immune Response of Nile Tilapia (*Oreochromis niloticus*). *BMC Veterinary Research*.

[22] Zafar N., Khan M. A. (2024). Effects of Dietary Zinc on Growth, Haematological Indices, Digestive Enzyme Activity, Tissue Mineralization, Antioxidant and Immune Status of Fingerling *Heteropneustes fossilis*. *Biology and Trace Elements Research*.

[23] Kou H., Xu S., Wang A.-L. (2015). Effect of Replacing Canola Meal for Fish Meal on the Growth, Digestive Enzyme Activity, and Amino Acids, of Ovate *Pompano*, *Trachinotus ovatus*. *Israeli Journal of Aquaculture-Bamid*.

[24] El-Araby D. A., Amer S. A., Attia G. A. (2022). Dietary Spirulina Platensis Phycocyanin Improves Growth, Tissue Histoarchitecture, and Immune Responses, With Modulating Immunoexpression of CD3 and CD20 in Nile Tilapia, *Oreochromis niloticus*. *Aquaculture*.

[25] Liu H., Yang J. J., Dong X. H. (2020). Effects of Different Dietary Carbohydrate-to-Lipid Ratios on Growth, Plasma Biochemical Indexes, Digestive, and Immune Enzymes Activities of Sub-Adult Orange-Spotted Grouper *Epinephelus coioides*. *Fish Physiology and Biochemistry*.

[26] Huang S. C., Chen S. M., Huang C. H. (2010). Effects of Dietary Zinc Levels on Growth, Serum Zinc, Haematological Parameters and Tissue Trace Elements of Soft-Shelled Turtles, *Pelodiscus sinensis*. *Aquaculture Nutrition*.

[27] Liu F., Qu Y. K., Wang A. M. (2019). Effects of Carotenoids on the Growth Performance, Biochemical Parameters, Immune Responses and Disease Resistance of Yellow Catfish (*Pelteobagrus fulvidraco*) Under High-Temperature Stress. *Aquaculture*.

[28] Zhou C., Lin H., Huang Z., Wang J., Wang Y., Yu W. (2021). Effects of Dietary Zinc Levels on Growth Performance, Digestive Enzyme Activities, Plasma Physiological Response, Hepatic Antioxidant Responses and Metallothionein Gene Expression in Juvenile Spotted Sea Bass (*Lateolabrax maculatus*). *Aquaculture Nutrition*.

[29] Mondal A. H., Behera T., Swain P. (2020). Nano Zinc Vis-à-Vis Inorganic Zinc as Feed Additives: Effects on Growth, Activity of Hepatic Enzymes and Non-Specific Immunity in Rohu, *Labeo rohita* (Hamilton) Fingerlings. *Aquaculture Nutrition*.

[30] Liang X. F., Cao C. Y., Chen P. Effects of Dietary Zinc Sources and Levels on Growth Performance, Tissue Zinc Retention and Antioxidant Response of Juvenile Common Carp (*Cyprinus carpio var*. Jian) Fed Diets Containing Phytic Acid. *Aquaculture Nutrition*.

[31] Zoli M., Rossi L., Bibbiani C., Bacenetti J. (2023). Life Cycle Assessment of Seabass and Seabream Production in the Mediterranean Area: A Critical Review. *Aquaculture*.

[32] Huang Q. C., Wang E. L., Dong X. H. (2018). Investigations on Zinc Bioavailability of Different Sources and Dietary Zinc Requirement in Juvenile Grouper *Epinephelus coioides*. *Aquaculture Research*.

[33] Montero D., Moyano J. F., Carvalho M. (2023). Nutritional Innovations in Superior Gilthead Seabream (*Sparus aurata*) Genotypes: Implications in the Utilization of Emerging New Ingredients Through the Study of the Patterns of Secretion of Digestive Enzymes. *Aquaculture*.

[34] Swain P. S., Rao S. B. N., Rajendran D., Dominic G., Selvaraju S. (2016). Nano Zinc, an Alternative to Conventional Zinc as Animal Feed Supplement: A Review. *Animal Nutrition*.

[35] Muralisankar T., Bhavan P. S., Radhakrishnan S., Seenivasan C., Manickam N., Srinivasan V. (2014). Dietary Supplementation of Zinc Nanoparticles and Its Influence on Biology Physiology and Immune Responses of the Freshwater Prawn *Macrobrachium rosenbergii*. *Biology and Trace Elements Research*.

[36] Muralisankar T., Bhavan P. S., Radhakrishnan S., Seenivasan C., Srinivasan V., Santhanam P. (2015). Effects of Dietary Zinc on the Growth, Digestive Enzyme Activities, Muscle Biochemical Compositions, and Antioxidant Status of the Giant Freshwater Prawn *Macrobrachium rosenbergii*. *Aquaculture*.

[37] Hu C. H., Xiao K., Jiao L. F., Song J. (2014). Effects of Zinc Oxide Supported on Zeolite on Growth Performance, Intestinal Barrier Function and Digestive Enzyme Activities of Nile Tilapia. *Aquaculture Nutrition*.

[38] Dawood M. A. O., Amer A. A., Gouda A. H., Gewaily M. S. (2023). Interactive Effects of Cyclical Fasting, Refeeding, and Dietary Protein Regimes on the Growth Performance, Blood Health, and Intestinal Histology of Nile Tilapia (*Oreochromis niloticus*). *Aquaculture*.

[39] Mohtashemipour H., Mohammadian T., Mozanzadeh T. M., Mesbah M., Nejad J. A. (2024). Dietary Selenium Nanoparticles Improved Growth and Health Indices in Asian Seabass (*Lates calcarifer*) Juveniles Reared in High Saline Water. *Aquaculture Nutrition*.

[40] Kumar N., Krishnani K. K., Singh N. P. (2018). Effect of Dietary Zinc-Nanoparticles on Growth Performance, Anti-Oxidative and Immunological Status of Fish Reared Under Multiple Stressors. *Biology and Trace Elements Research*.

[41] Kumari K., Sahoo S., Khumujam S. D. (2025). Effect of Chronic Crowding Stress on Haematology, Serum Biochemistry and Scale Cortisol Levels of *Labeo rohita* (Hamilton, 1822) Fingerlings. *Aquaculture*.

[42] Li M.-Y., Liu Y.-Z., Chen X.-M. (2025). Astaxanthin Ameliorates High-Carbohydrate Diet-Induced ER Stress, Immunosuppression and Hepatic Glucose Metabolism Through AMPK/Autophagy Pathway in *Channa argus*. *Aquaculture*.

[43] Jiang M., Wu F., Huang F. (2016). Effects of Dietary Zn on Growth Performance, Antioxidant Responses, and Sperm Motility of Adult Blunt Snout Bream, *Megalobrama amblycephala*. *Aquaculture*.

[44] Peng D., Yang L., Liang X.-F., Chai F. (2023). Dietary Zinc Levels Affect Growth, Appetite, and Lipid Metabolism of Chinese Perch (*Siniperca chuatsi*). *Fish Physiology and Biochemistry*.

[45] Jiang M., Huang F., Wen H. (2015). Effects of Dietary Zinc on Growth, Serum Biochemical Indexes and Antioxidant Responses of Juvenile Blunt Snout Bream, *Megalobrama Amblycephala*. *Journal of Fishery Sciences of China*.

[46] Yuan Y., Luo J., Zhu T. (2020). Alteration of Growth Performance, Meat Quality, Antioxidant and Immune Capacity of Juvenile *Litopenaeus vannamei* in Response to Different Dietary Dosage Forms of Zinc: Comparative Advantages of Zinc Amino Acid Complex. *Aquaculture*.

[47] Xia Y., Wang Y., Xie J. (2022). Effects of BBR on Growth Performance, Serum and Hepatic Biochemistry Parameters, Hepatic Morphology and Gene Expression Levels Related to Glucose Metabolism in Largemouth Bass, *Micropterus salmoides*. *Aquaculture Research*.

[48] Banaee M., Gholamhosseini A., Sureda A., Soltanian S., Fereidouni M. S., Ibrahim A. T. A. (2021). Effects of Microplastic Exposure on the Blood Biochemical Parameters in the Pond Turtle (*Emys orbicularis*). *Environmental Science & Pollution Research*.

[49] Chaklader M. R., Ahmed H. A., Khafaga A. F., Shukry M., Abo Selema T. A. M., Abdel-Latif H. M. R. (2024). *Silybum marianum* Promotes Growth, Hepatic Antioxidative Activity, and Splenic Immunity but Does Not Influence the Intestinal Barrier Function of Nile Tilapia, *Oreochromis niloticus*. *Aquaculture*.

[50] Mohseni M., Hamidoghli A., Bai S. C. (2021). Organic and Inorganic Dietary Zinc in Beluga Sturgeon (*Huso huso*): Effects on Growth, Hematology, Tissue Concentration and Oxidative Capacity. *Aquaculture*.

[51] Pan S., Yan X., Tan B. (2022). Effects of Dietary Zinc Sources and Levels on Growth Performance, Serum Biochemical and Immunological Indexes and Tissue Zinc Content of *Litopenaeus vannamei*. *Aquaculture Reports*.

[52] Li M.-R., Huang C.-H. (2016). Effect of Dietary Zinc Level on Growth, Enzyme Activity and Body Trace Elements of Hybrid Tilapia, *Oreochromis niloticus* × *O. aureus*, Fed Soya Bean Meal-Based Diets. *Aquaculture Nutrition*.

[53] Yu H. R., Li L. Y., Shan L. L., Gao J., Ma C. Y., Li X. (2021). Effect of Supplemental Dietary Zinc on the Growth, Body Composition and Anti-Oxidant Enzymes of Coho Salmon (*Oncorhynchus kisutch*) Alevins. *Aquaculture Research*.

[54] Wang N., Wang X., Lin Z. (2022). Effects of Dietary Zn on Growth, Antioxidant Capacity, Immunity and Tolerance to Lipopolysaccharide Challenge in Juvenile Chinese Mitten Crab *Eriocheir sinensis*. *Aquaculture Research*.

[55] Zhang M., Huang Y., Li Y., Cai M., Zhao Y. (2021). The Effects of Dietary Zinc on Growth, Immunity and Reproductive Performance of Female *Macrobrachium nipponense* Prawn. *Aquaculture Research*.

[56] Raibeemol K. P., Chitra K. C. (2020). Induction of Immunological, Hormonal and Histological Alterations After Sublethal Exposure of Chlorpyrifos in the Freshwater Fish, *Pseudetroplus maculatus* (Bloch, 1795). *Fish & Shellfish Immunology*.

[57] Huang M., Wei X., Wu T. (2022). Inhibition of TNBS-Induced Intestinal Inflammation in Crucian Carp (*Carassius carassius*) by Oral Administration of Bioactive Food Derived Peptides. *Fish & Shellfish Immunology*.

[58] Sukkarun P., Kitiyodom S., Kamble M. T. (2024). Systemic and Mucosal Immune Responses in Red Tilapia (*Oreochromis sp*.) Following Immersion Vaccination With a Chitosan Polymer-Based Nanovaccine Against *Aeromonas veronii*. *Fish & Shellfish Immunology*.

[59] Cui Z., Zhao H., Chen X. (2023). Molecular and Functional Characterization of Two IgM Subclasses in Large Yellow Croaker (*Larimichthys crocea*). *Fish & Shellfish Immunology*.

[60] Song Z. X. (2017). *Effects of Dietary Zinc on the Growth Performance, Intestinal Health, Body Health, Gill Health and Flesh Quality, as Well as the Mechanisms in Young Grass Carp (Ctenopharyngodon idella)*.

[61] Zhou W., Yang S., Huang K. (2024). Can *Chilodonella uncinata* Induce Cross-Protection in Koi Carp (*Cyprinus carpio*) Against *Ichthyophthirius multifiliis*? Evidence From Immune Response and Challenge Experiments. *Aquaculture*.

[62] Zhuo M. Q., Chen X., Gao L. (2023). Early Life Stage Exposure to Cadmium and Zinc Within Hour Affected GH/IGF Axis, Nrf2 Signaling and HPI Axis in Unexposed Offspring of Marine Medaka *Oryzias melastigma*. *Aquatic Toxicology*.

[63] Kishawy A. T. Y., Roushdy E. M., Hassan F. A. M., Mohammed H. A., Abdelhakim T. M. N. (2020). Comparing the Effect of Diet Supplementation With Different Zinc Sources and Levels on Growth Performance, Immune Response and Antioxidant Activity of Tilapia, *Oreochromis niloticus*. *Aquaculture Nutrition*.

[64] Vistro W. A., Zhang Y., Azhar M. (2020). Hematological and Plasma Biochemical Parameters of Chinese Soft-Shelled Turtle During Hibernation and Non-Hibernation. *International Journal of Agricultural and Biological Engineering*.

[65] Horie Y., Yonekura K., Suzuki A., Takahashi C. (2020). Zinc Chloride Influences Embryonic Development, Growth, and Gh/Igf-1 Gene Expression During the Early Life Stage in Zebrafish (*Danio rerio*). *Comparative Biochemistry and Physiology Part C: Toxicology & Pharmacology*.

